# Extrapleural Superficial Solitary Fibrous Tumor on the Posterior Shoulder: A Case Report and Review of the Literature

**Published:** 2018-10-11

**Authors:** Sean J. Wallace, Robert Teixeira, Nathan F. Miller, Mamtha Raj, Hina Sheikh, Rohit Sharma

**Affiliations:** ^a^Division of Plastic & Reconstructive Surgery, Department of Surgery, Lehigh Valley Health Network, Allentown, Pa; ^b^Division of Dermatopathology, Department of Pathology, Health Network Laboratories, Allentown, Pa; ^c^Lehigh Valley Physicians Group–Surgical Oncology, Allentown, Pa

**Keywords:** solitary fibrous tumor, superficial solitary fibrous tumor, extrapleural solitary fibrous tumor, CD34, STAT6

## Abstract

**Objective**: Mesenchymal in origin, solitary fibrous tumors are primarily seen within the pleura of the lung or in serosal-lined body cavities. Constituting 1% to 2% of all soft-tissue tumors, solitary fibrous tumors are rare entities, especially when found in extrapleural and in superficial locations. A review of *PubMed MEDLINE* literature for superficial solitary fibrous tumors revealed 71 reports in case reports and small case series. **Methods**: In this report, we describe a 74-year-old man with an extrapleural superficial solitary fibrous tumor, as well as present a review of the current published literature to date. **Results**: We present the clinical course, surgical procedure, histopathological features, and treatment options, as well as present a compilation of the published data on superficial solitary fibrous tumors. **Conclusions**: Based on the current literature, solitary fibrous tumors are more common in middle-aged women and in the head and neck region. Diagnosis of solitary fibrous tumors requires tissue sampling and staining for immunohistochemical markers. Management of these tumors is based on wide local excision with histologically negative margins. If negative margins cannot be surgically achieved, adjuvant therapies including radiation have been described. With extrapleural manifestations of solitary fibrous tumors seldom reported in the literature, it is our hope that reporting these unusual instances will raise awareness of such disease manifestations and allow for earlier diagnosis and treatment.

A spindle-cell neoplasm of mesenchymal origin, solitary fibrous tumors (SFTs) were first reported in 1931.^1^ Previously referred to as hemangiopericytomas, SFTs are primarily seen within deep soft tissue, notably in the pleura of the lung and/or on serosal surfaces. They tend to affect adults between the fourth and seventh decades of life.^1^ Grossly, these tumors are often well-circumscribed, mobile, and painless. Histologically, these tumors are found to have alternating areas of hyper- and hypocellularity, variable concentrations of collagen, and characteristic gaping and bifurcating (staghorn) vessels. Immunohistochemical markers, such as *CD34* and *STAT6*, have been increasingly utilized in narrowing down the diagnosis. The biological course of SFTs tends be benign in nature, but malignant potential does exist. Clinically, patients may experience associated hypoglycemia, arthralgias, osteoarthritis, and clubbing. SFTs found in the cutis and subcutis are classified as superficial. Extrapleural superficial SFTs are quite rare but have been previously described around the body including both soft tissue and viscera with a tendency to present in a body cavity.

Worldwide, approximately 850 cases of SFTs have been reported in the medical literature.^1^ A review of *PubMed MEDLINE* involving repots of superficial SFTs (cutaneous/subcutaneous) utilizing search terms (solitary fibrous tumor [Title/abstract]) AND (skin OR subcutaneous OR cutaneous OR superficial) revealed 71 cases having been identified and described in the cutis and subcutis as case reports and/or small case series (Table 1).

Diagnosis of SFTs requires sampling of the tumor with subsequent histopathological examination. Techniques including cytogenic analysis and immunohistochemical (IHC) staining can identify expression of *CD34*, *bcl-2*, *CD99*, and nuclear fusion gene *STAT6*, which are reliable surrogates for detection and diagnosis of SFTs.^27^ Approximately 15% to 20% of these neoplasms may go on to develop metastatic potential with hematogenous dissemination, most commonly to the lungs. Concerning histological features for recurrence and metastasis include diameter greater than 5 cm, significant pleomorphism, atypia, high cellularity, mitotic figures more than 4/10 high-powered field (HPF), and tumor cell necrosis.^28^

Management of SFTs requires appropriate staging and evaluation for metastases with computed tomography. Benign tumors can be managed with wide local excision, with an expected 5-year survival ranging from 89% to 100%.^29^ Metastatic tumors may require radiation therapy or antiangiogenic therapies that target vascular endothelial growth factor. There is no strong evidence to show that chemotherapy has increased survivability in the management of SFTs.^29^

Overall, occurrence of SFTs is low, representing about 1% to 2% of all soft-tissue tumors.^29^ Extrapleural SFTs are even less common, representing 0.6% of all soft-tissue tumors.^20^^,^^29^ The majority of patients with SFTs have a relatively benign course; however, because of their malignant potential, they require long-term follow-up. In this report, we describe an instance of an unusual case of SFT presenting in the soft tissue of the posterior shoulder. We present the clinical, surgical, and histopathological features, as well as discuss the treatment options and review the published medical literature on superficial SFTs.

## CASE REPORT

A 74-year-old man was evaluated by dermatology for a suspicious painless mass located over the posterior aspect of his right trapezius muscle. The mass had been present for at least 3 years but was noted to have had a rapid increase in size within the last 2 months prior to presentation. He denied any associated symptoms or recent changes in his health. Medical and surgical histories were significant for actinic keratoses, atrial fibrillation with cardiac ablation and placement of a permanent pacemaker, coronary artery disease, hypertension, hyperlipidemia, and benign prostatic hypertrophy. He denied any family history of malignancy, other than actinic keratoses. On examination, he was noted to have a palpable, nontender, mobile mass over the posterior aspect of his right upper trapezius muscle measuring approximately 3 × 3 cm. An incisional biopsy was performed by dermatology. Pathology revealed cellular spindle cell tumor without necrosis but with up to 6 mitoses/10 HPF. In addition, IHC staining was positive for *CD34* and *CD99*. The histopathology was reviewed and the diagnosis of SFT was confirmed by the Department of Pathology by performing IHC staining for *STAT6*.

The patient was then seen in the surgical oncology office for further discussion and management. He was found to have a 3.5 × 1.2-cm mass with an overlying healing scar from his incisional biopsy. There was no evidence of satellitosis. Computed tomography of the chest, abdomen, and pelvis was performed to determine the extent of the tumor and revealed no evidence of metastatic disease. Prior to wide local excision, the patient was evaluated by the Multidisciplinary Cutaneous Oncology Clinic for any additional treatment recommendations. Neoadjuvant therapies were not recommended.

A full-thickness, wide local excision with 1-cm margins was performed. Additional trapezius muscle was taken for an oncological boundary of safety. All specimens were submitted to pathology. The primary resection defect measured 6.1 × 5.4 × 3.6 cm and was reconstructed with a local rotational-advancement flap.

Postoperatively, the flap reconstruction healed well without complication. Permanent pathology revealed positive deep margins with residual SFT. After extensive discussion with the patient, the decision was made to pursue adjuvant radiation therapy and forgo a secondary surgery. Radiation oncology plans for 30 treatments.

## DISCUSSION

SFTs are of mesenchymal origin, most often located within the pleura. Extrapleural manifestations represent about 0.6% of all SFTs, with the most common location being the head and neck.^20^ Previously described locations for extrapleural SFTs include the lumbar extradural space, intrameningeal space, cervical spine, deep soft tissue of the neck, orbital space, pelvic space, retroperitoneal space, vagina, thyroid gland, mammary gland, prostate, nasal mucosa, liver, renal pelvis, and the extremities.^1^ Here, we present and discuss an extrapleural superficial SFT overlying the trapezius muscle.

Summarized in Table 1 is a compilation of superficial SFTs published to date. From this review, women are found to be the most common gender affected, while the most frequent anatomic location is the head and neck region. The mean age of the reported cases is 43.9 years (range, 8 months-88 years). The average size is 5.2 cm (range, 0.4-15.5 cm). After wide local excision, the majority of patients went on to be disease free. Imunohistochemically, *CD34* expression was found to be more commonly reported than any other molecular marker. Underreporting of *STAT6* in the literature may be secondary to more recent understanding and evidence of expression of *NAB2-STAT6* translocation and gene fusion that is now utilized for confirmation of diagnosis.^30^

**Figure 1:**
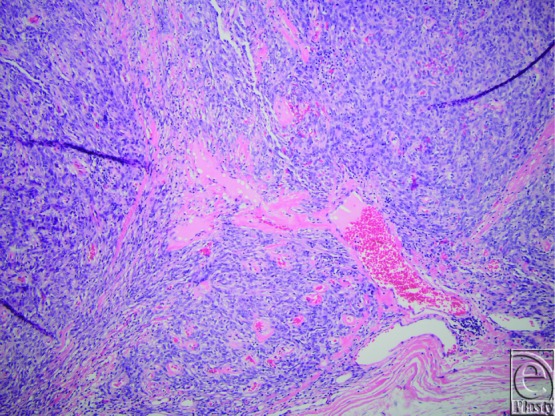
A representative routine hematoxylin-eosin section at 10× magnification showing hypo- and hypercellular areas of blood vessels and prominent perivascular growth of tumor.

**Figure 2:**
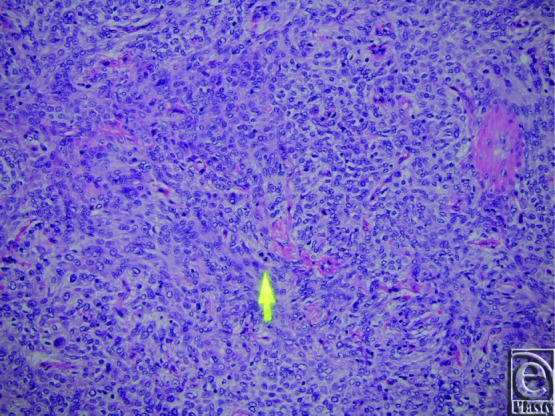
A representative immunohistochemical stain at 10× magnification showing the positivity for *CD34*.

**Figure 3:**
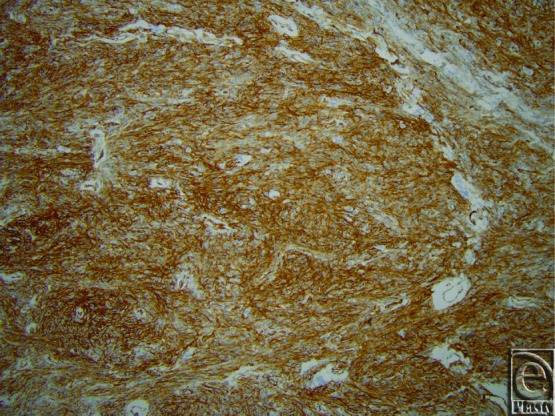
A representative immunohistochemical stain at 10× magnification showing the positivity for *STAT6*.

**Figure 4:**
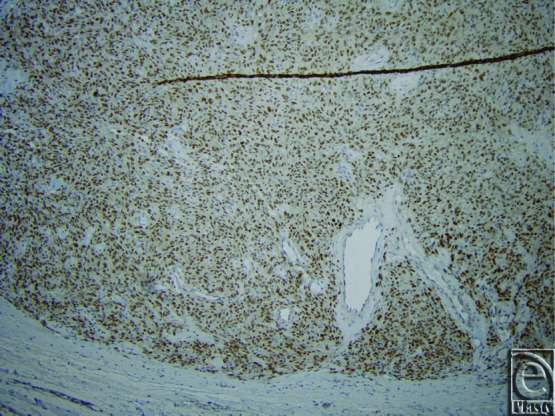
A representative routine hematoxylin-eosin section at 20× magnification showing mitosis and low-grade atypia.

Pathological diagnosis of SFTs requires recognition of histological features coupled with supportive IHC stains. Similar to their pleural counterparts, this is a spindle cell neoplasm with alternating areas of hyper- and hypocellularity and characteristic gaping and bifurcating vessels, also known as staghorn vessels (Fig 1). Hyalinization of the vessels and prominent perivascular hemangiopericytoma-like pattern of growth are subtle features to establishing the diagnosis. Cellular variants of SFTs in the past were classified as hemangiopericytoma but are now incorporated into the spectrum of SFTs. IHC staining that is *CD34* positive (Fig 2) is indicative of perivascular cells, the putative cell of origin of this tumor. *CD99* and *bcl-2* can also show variable staining, but these stains are not lineage specific. Recently, a recurrent paracentric inversion involving chromosome 12q13 has been identified in SFTs of pleura and soft tissue that result in *NAB2-STAT6* translocation and gene fusion.^30^ This gene fusion results in oncogenic overexpression of activation factor *STAT6* that drives tumor proliferation. *STAT6* immunostain is reliable surrogate marker for this molecular change and was employed for confirmation of the diagnosis in this case (Fig 3). Strong criteria for malignancy have not been previously well-described in this tumor, but risk stratification models have been proposed. Concerning histological features for recurrence and metastasis include significant size greater than 5 cm, pleomorphism, atypia, high cellularity, mitotic figures more than 4/10 HPF, and tumor cell necrosis^28^ (Fig 4). Age of patient, tumor size, and mitotic activity have shown discriminatory power in separating tumors into low, intermediate, and high risk for recurrence/metastasis in a cohort of approximately 100 patients.^31^

Extrapleural location of SFTs does not necessarily portend a higher rate of metastasis/recurrence; however, its behavior is generally unpredictable. Reported recurrence and metastasis rates range from 10% to 37% in the literature.^32^ Positive margins after resection are reported to threaten a higher risk of metastasis and recurrence.^32^^,^^33^ Recommended treatment is wide local excision of the tumor and its capsule. Failure to fully remove the capsule has a higher rate of local recurrence, but the risk in this case can be minimized with adjuvant therapy.^33^ If a patient remains with positive margins postoperatively, radiation therapy and/or antiangiogenic inhibitors may be considered. In this case, the patient was found to have positive margins after initial resection but he elected to forego additional surgery and pursue adjuvant radiation therapy. Long-term follow-up is recommended and will be provided.

With extrapleural superficial SFTs seldom reported in the literature, it is our hope that reporting our experience will add to the database of published literature, raise awareness of extrapleural manifestations, and allow for earlier diagnosis and treatment.

## Figures and Tables

**Table 1:** Review of *PubMed MEDLINE* literature involving case reports and case series of superficial SFTs

								*STAT6*	*CD34*	
Reference	Year	PMID	Journal	Location	Sex	Age, y	Size, cm	expression	expression	Outcome
Feasel et al^2^	2018	29438169	*The American Journal of Surgical Pathology*	Head, thigh, back, shoulder, upper arm, ankle, toe	F 16: M 7	46 (16-80)	2.9 (1.0-7.0)	17/18 positive	21/22 positive	Disease free
Zhao et al^3^	2018	29325251	*Chinese Journal of Pathology*	Head/neck soft tissue ×3, 2 subcutaneous trunk	Not reported	39 (23-54)	3.1 (0.4-8.0)	Positive	Not reported	Not reported
Pearre et al^4^	2017	29201988	*Gynecologic Oncology Reports*	Vulva	F	64	9	Not reported	Positive	Death from disease at 15 mo
Lee^5^	2016	27352579	*European Journal of Gynaecological Oncology*	Mons pubis	F	57	9	Not reported	Positive	Disease free
Creytens et al^6^	2016	27062638	*Journal of Cutaneous Pathology*	Skin	F	64	3	Positive	Positive	Disease free
Lee et al^7^	2016	25979291	*Journal of Foot and Ankle Surgery*	Ankle	F	69	0.7	Not reported	Positive	Disease free
Yoshimura et al^8^	2016	26967903	*International Journal of Surgical Case Reports*	Thigh	M	31	13	Not reported	Positive	Malignant recurrence at 11 mo; reexcised and disease free at the time of report publication
Lee et al^9^	2015	25140663	*The American Journal of Dermatopathology*	Palm	F	46	1	Not reported	Positive	Disease free
Tenekeci et al^10^	2015	26102546	*Journal of Craniofacial Surgery*	Intraorbital	M	51	9.5	Not reported	Positive	Not reported
Kishimoto et al^11^	2014	25946830	*Nihon Jibiinkoka Gakkai Kaiho*	Intraorbital	M	75	3.8	Not reported	Positive	disease free
Satomi et al^12^	2014	24221815	*Medical Molecular Morphology*	Cheek	M	47	8	Not reported	Positive	Disease free
Soriano-Hernandez et al^13^	2014	25238475	*Cirugia y Cirujanos*	Finger	M	43	2.5	Not reported	Positive	disease free
Rizk et al^14^	2013	23140216	*Journal of Neurosurgery: Pediatrics*	Scalp	M	2	Not reported	Not reported	Positive	Disease free
Terada^15^	2011	21244389	*International Journal of Dermatology*	Shoulder	F	49	8	Not reported	Positive	Disease free
Tsirevelou et al^16^	2010	20868476	*Head & Face Medicine*	Neck	F	74	9	Not reported	Positive	Disease free
Wood et al^17^	2010	20559119	*The American Journal of Dermatopathology*	Thigh ×3, lower extremity ×2, abdomen	F 4: M 2	55 (25-88)	Not reported	Not reported	Positive	Not reported
Tourabi et al^18^	2008	18550249	*Annales de Chirurgie Plastique Esth*é*tique*	Scalp	M	47	8	Not reported	Positive	Disease free
Soldano and Meehan^19^	2008	18212546	*The American Journal of Dermatopathology*	Abdomen, glabella	F	26, 35	1.5	Not reported	Positive	Disease free
Erdag et al^20^	2007	17944724	*Journal of Cutaneous Pathology*	Scalp, toe, cheek ×2, back ×2, lip, forehead, heel, temple	F 2: M 8	43.5 (8-61 mo)	1.2 (0.8-2.5)	Not reported	8/10 positive	Multiple recurrences for the 8-mo-old but now disease free at 8 y; other cases disease free (n = 7) or not reported (n = 2)
Matsushita et al^21^	2005	16471474	*The Journal of Dermatology*	Perioral	M	34	1.5	Not reported	Positive	Disease Free
Yoshida et al^22^	2004	15801268	*The Journal of Dermatology*	Back	F	56	4	Not reported	Positive	Disease free
Hardisson et al^23^	2002	11807468	*Journal of the American Academy of Dermatology*	Cheek	F	56	1.5	Not reported	Positive	Disease free
Ramdial and Madaree^24^	2001	11370264	*Pediatric and Developmental Pathology*	Scalp	F	1	15.5	Not reported	Positive	Disease free
Cowper et al^25^	1999	10380040	*The American Journal of Dermatopathology*	Neck ×2, occiput	F 1: M 2	46, 38, 63	3,3,4	Not reported	Positive	Disease free
Okamura et al^26^	1997	9335244	*The American Journal of Dermatopathology*	Scalp	F	37	Not reported	Not reported	Positive	Disease free
